# Vaccination with Enzymatically Cleaved GPI-Anchored Proteins from *Schistosoma mansoni* Induces Protection against Challenge Infection

**DOI:** 10.1155/2012/962538

**Published:** 2012-08-15

**Authors:** Vicente P. Martins, Carina S. Pinheiro, Barbara C. P. Figueiredo, Natan R. G. Assis, Suellen B. Morais, Marcelo V. Caliari, Vasco Azevedo, William Castro-Borges, R. Alan Wilson, Sergio C. Oliveira

**Affiliations:** ^1^Departamento de Bioquímica, Imunologia do Instituto de Ciências Biológicas, Universidade Federal de Minas Gerais, 31270-901 Belo Horizonte, MG, Brazil; ^2^Instituto Nacional de Ciência e Tecnologia em Doenças Tropicais (INCT-DT), CNPq MCT, 40110-160 Salvador, BA, Brazil; ^3^Departamento de Biologia Geral do Instituto de Ciências Biológicas, Universidade Federal de Minas Gerais, 31270-901 Belo Horizonte, MG, Brazil; ^4^Departamento de Patologia Geral do Instituto de Ciências Biológicas, Universidade Federal de Minas Gerais, 31270-901 Belo Horizonte, MG, Brazil; ^5^Departamento de Ciências Biológicas, Universidade Federal de Ouro Preto, 35400-000 Ouro Preto, MG, Brazil; ^6^Centre for Immunology & Infection, Department of Biology, University of York, Heslington, York YO10 5DD, UK

## Abstract

The flatworm *Schistosoma mansoni* is a blood fluke parasite that causes schistosomiasis, a debilitating disease that occurs throughout the developing world. Current schistosomiasis control strategies are mainly based on chemotherapy, but many researchers believe that the best long-term strategy to control schistosomiasis is through immunization with an antischistosomiasis vaccine combined with drug treatment. In the search for potential vaccine candidates, numerous tegument antigens have been assessed. As the major interface between parasite and mammalian host, the tegument plays crucial roles in the establishment and further course of schistosomiasis. Herein, we evaluated the potential of a GPI fraction, containing representative molecules located on the outer surface of adult worms, as vaccine candidate. Immunization of mice with GPI-anchored proteins induced a mixed Th1/Th2 type of immune response with production of IFN-**γ** and TNF-**α**, and low levels of IL-5 into the supernatant of splenocyte cultures. The protection engendered by this vaccination protocol was confirmed by 42% reduction in worm burden, 45% reduction in eggs per gram of hepatic tissue, 29% reduction in the number of granulomas per area, and 53% reduction in the granuloma fibrosis. Taken together, the data herein support the potential of surface-exposed GPI-anchored antigens from the *S. mansoni* tegument as vaccine candidate.

## 1. Introduction

Schistosomiasis mainly occurs in developing countries and it is the most important human helminth infection in terms of global mortality. This parasitic disease affects more than 200 million people worldwide causing more than 250,000 deaths per year [1]. Furthermore, schistosomiasis causes up to 4.5 million DALY (disability adjusted life year) losses annually [2]. Current schistosomiasis control strategies are mainly based on chemotherapy but, in spite of decades of mass treatment, the number of infected people remains constant [3]. Extensive endemic areas and constant reinfection of individuals together with poor sanitary conditions in developing countries make drug treatment alone inefficient [4]. Many consider that the best long-term strategy to control schistosomiasis is through immunization with an antischistosomiasis vaccine combined with drug treatment [5]. A vaccine that induces even a partial reduction in worm burdens could considerably reduce pathology and limit parasite transmission [6].

Currently, the most promising schistosome vaccine candidates are located in the tegument of the worms [7], such as TSP-2 [8] and Sm29 [9]. The tegument is a dynamic host-interactive surface involved in nutrition, immune evasion/modulation, excretion, osmoregulation, sensory reception, and signal transduction [10, 11]. The outer surface of this major parasite/host interactive surface is rich in GPI-anchored proteins [12], as well as other antigens such as aquaporin, phosphohydrolases, annexin-2, and Sm200 [13]. Sm200 and Sm29 are among the most abundant GPI-anchored proteins in the *S. mansoni* tegument surface [12, 13]. Even with unknown function, these two proteins are intriguing because their high levels of expression in the schistosomulum and adult stages imply that they are certainly playing important roles in the parasite host interaction.

The glycosylphosphatidylinositol (GPI) anchor is a posttranslational modification that anchors the carboxi-terminus of modified proteins in the outer leaflet of the cell membrane [14–17]. GPI-anchored proteins comprise molecules with a variety of functions and structures playing central roles in biological systems, such as signal transduction, immune responses, and the pathophysiology of some pathogenic diseases [18]. Given that the *S. mansoni* outer surface is loaded with GPI-anchored proteins, it is reasonable to assume that these molecules are involved in host/parasite interactions, placing them as potential targets for immune and chemotherapeutic treatment of schistosomiasis.

The use of preparations containing tegument proteins as antigens to immunize and protect mice was earlier shown to be feasible [19]. Herein, we evaluated the potential of GPI-anchored proteins of *S. mansoni* to elicit an immunological response able to protect mice against cercarial challenge and reduce the pathology associated with schistosomiasis.

## 2. Materials and Methods

### 2.1. Ethics Statement

Animal experiments were conducted in accordance with the Brazilian Federal Law number 11.794 which regulates the scientific use of animals and IACUC guidelines. All protocols were approved by the Committee of Ethics for Animal Experimentation (CETEA) at UFMG under permit 179/2010.

### 2.2. Mice and Parasites

Female C57BL/6 or Swiss mice aged 6–8 weeks were purchased from the Federal University of Minas Gerais (UFMG) animal facility. Cercariae of *S. mansoni* (LE strain) was maintained routinely on *Biomphalaria glabrata* snails at CPqRR (Centro de Pesquisa René-Rachou-Fiocruz) and prepared by exposing infected snails to light for 1 h to induce shedding. Cercarial numbers and viability were determined using a light microscope prior to infection.

### 2.3. Phosphatidylinositol-Phospholipase C (PiPLC) Treatment of Live Parasites

GPI-anchored proteins were recovered from live worms by *in vitro* incubation with PiPLC as the shaving enzyme, following the protocol previously described [12]. The experiment was performed twice to provide biological replicates. Downstream processing utilized the worms from 60 Swiss mice previously infected with 200 cercariae. Mice were perfused and treated in two separate batches to minimize the time *ex vivo*, supernatants were then combined. Each batch was incubated at 37°C for 1 h in the presence of PiPLC (from *Bacillus cereus*, Sigma-Aldrich, St. Louis, Mo USA) at 1.25 Units/mL, in a 30 mL Corning flask (Corning, NY, USA) containing 5 mL of buffered RPMI. The supernatant was recovered, transferred to a 15 mL Falcon tube, and centrifuged at 500 ×g for 30 min to remove any insoluble material such as the haematin particles in gut vomitus. The supernatant was removed and concentrated at 4°C using a 5,000 Da cut-off centrifugation device (Vivaspin 6, West Sussex, UK). The control for secretion, vomitus production, and parasite damage due to handling comprised an identical experiment, minus PiPLC.

### 2.4. SDS-PAGE and Immunoblotting

SDS-PAGE of purified GPI-anchored proteins was performed [20]. The gel was electroblotted onto nitrocellulose membrane (Bio-Rad, Hercules, Calif, USA) [21]. Membrane was blocked with TBS-T (0.5 M NaCl−0.02 M Tris (pH 7.5), 0.05% Tween 20) containing 5% nonfat dry milk for 16 hrs at 4°C. Subsequently, the membrane was incubated in a 1 : 1,000 dilution of anti-Sm29 or anti-Sm200 murine polyclonal antibodies for 1 hr at room temperature. After three washes using TBS-T the membrane was incubated in 1 : 2,000 goat anti-mouse IgG conjugated with alkaline phosphatase (AP) (Invitrogen, Carlsbad, Calif, USA), treated with AP reaction mixture in a developing buffer containing nitroblue tetrazolium (NBT) and 5-bromo-4-chloro-3-indolyl-1-phosphate (BCIP, Invitrogen, Carlsbad/CA, USA). After the reaction was developed, the membrane was washed using distilled water and dried on filter paper.

### 2.5. Immunization of Mice

Six-to-eight-week-old female C57BL/6 mice were divided into two groups of ten mice each. Mice were subcutaneously injected in the nape of the neck with 12.5 *μ*g of GPI-anchored proteins or PBS as a control on days 0, 15 and 30. We considered that Sm200 and Sm29 correspond to 87% (10.89 mg) and 0.44% (0.05 mg), respectively, of the total amount of GPI-anchored proteins [13]. The protein mixture was formulated with Complete Freund's Adjuvant (CFA, Sigma-Aldrich, St. Louis/MO, USA) for the first immunization and Incomplete Freund's Adjuvant (IFA, Sigma-Aldrich, St. Louis, Mo, USA) for the last two immunizations.

### 2.6. Challenge Infection and Worm Burden Recovery

Fifteen days after the last boost, mice were challenged by percutaneous exposure of abdominal skin for 1 h in water containing 100 cercariae (LE strain). Forty-five days after challenge, adult worms were perfused from the portal veins [22]. Two independent experiments were performed to determine protection levels. The protection was calculated by comparing the number of worms recovered from each vaccinated group with its respective control group, using the formula:

(1) PL  =  WRCG−WREG×100WRCG,

where PL = protection level, WRCG = worms recovered from control group, and WREG = worms recovered from experimental group.

### 2.7. Measurement of Specific Antibodies

Following immunization, sera of ten mice from each vaccinated or control group were collected at two-week intervals. Measurements of specific antibodies were performed using indirect ELISA. Maxisorp 96-well microtiter plates (Nunc, Denmark) were coated with 5 *μ*g/mL of GPI-anchored proteins or with 5 *μ*g/mL of a mixture containing rSm200 and rSm29 in carbonate-bicarbonate buffer, pH 9.6 for 16 hrs at 4°C, then blocked for 2 hrs at room temperature with 200 *μ*L/well PBS-T (phosphate buffer saline, pH 7.2 with 0.05% Tween-20) plus 10% FBS (fetal bovine sera). One hundred microliters of each serum diluted 1 : 100 in PBS-T was added per well and incubated for 1 h at room temperature. Plate-bound antibodies were detected by peroxidase-conjugated goat anti-mouse IgG, IgG1, and IgG2a (Sigma-Aldrich, St. Louis/MO, USA) diluted in PBS-T 1 : 10000, 1 : 2000, and 1 : 2000, respectively. Color reaction was developed by addition of 100 *μ*L per well of 200 pmol OPD (o-phenylenediamine (Sigma-Aldrich, St. Louis, MO, USA) in citrate buffer, pH 5.0 plus 0.04% H_2_O_2_ for 10 min and stopped with 50 *μ*L of 5% sulfuric acid per well. The plates were read at 492 nm in an ELISA plate reader (Bio-Rad, Hercules, Calif, USA).

To measure specific IgE in the sera of vaccinated and control group, Maxisorp 96-well microtiter plates (Nunc, Denmark) were coated with 5 *μ*g/mL of a mixture containing rSm200 and rSm29 in carbonate-bicarbonate buffer, pH 9.6 overnight at 4°C, then blocked overnight at 4°C with 200 *μ*L/well PBS-T (phosphate buffer saline, pH 7.2 with 0.05% Tween-20) plus 3% non-fat dry milk. One hundred microliters of each serum diluted 1 : 50 in PBS-T was added per well and incubated overnight at 4°C. Followed by addition of 100 *μ*L of biotin-conjugated goat anti-mouse IgE (BD pharmingen, San Jose, Calif, USA) diluted 1 : 250 and incubated at room temperature for 1 h. Plate-bound antibodies were detected by adding 100 *μ*L of peroxidase-conjugated streptavidin (R&D Diagnostic, Minneapolis, Minn, USA) diluted 1 : 200 followed by 30 min of incubation at room temperature. Color reaction was developed by addition of 100 *μ*L per well of TMB (R&D Diagnostic, Minneapolis, Minn, USA) and incubated for 20 min, then stopped with 50 *μ*L of 5% sulfuric acid per well. The plates were read at 450 nm in an ELISA plate reader (Bio-Rad, Hercules, Calif, USA).

### 2.8. Cytokine Analysis

Cytokine experiments were performed using splenocyte cultures from individual mice immunized with GPI-anchored proteins or PBS as a control group (*n* = 4 for each group). Splenocytes were isolated from macerated spleen of individual mice one week after the third immunization, and washed twice with sterile PBS. After washing, the cells were adjusted to 1 × 10^6^ cells per well for IL-5, IL-10, IFN-*γ*, and TNF-*α* assays in RPMI 1640 medium (Invitrogen, Carlsbad, Calif, USA) supplemented with 10% FBS, 100 U/mL of penicillin G sodium, 100 *μ*g/mL of streptomycin sulfate, 250 ng/mL of amphotericin B. Splenocytes were maintained in culture with medium alone or stimulated with the GPI-anchored proteins (15 *μ*g/mL) or with concanavalin A (ConA) (5 *μ*g/mL) for IL-5, IL-10, IFN-*γ*, and LPS (1 *μ*g/mL) for TNF-*α*, as positive controls [22, 23]. The 96-well plates (Nunc, Denmark) were maintained in an incubator at 37°C with 5% CO_2_. Culture supernatants were collected after 24, 48, and 72 hrs. The assays for measurement of IL-5, IL-10, IFN-*γ*, and TNF-*α* were performed using the Duoset ELISA kit (R&D Diagnostic, Minneapolis, Minn, USA) according to the manufacturer's directions.

### 2.9. Histophathological Analysis

Following perfusion for the recovery of the schistosomes, liver sections from mice (8 animals per group) of control and experimental groups were collected to evaluate the effect of immunization in granuloma formation. The liver samples removed from the central part of the left lateral lobe were fixed with 10% buffered formaldehyde in PBS. Histological sections were performed using microtome at 5 *μ*m and stained on a slide with picrosirius. The area from each liver section (*μ*m^2^) was calculated using the KS300 software connected to a Carl Zeiss image analyzer. To perform measurements of the total area of granulomas, 20 granulomas with a single-well-defined egg, from each animal, were randomly chosen at a microscope with 10x objective lens. Granuloma images were obtained through a JVC TK-1270/RBG microcamera. Using a digital pad in the KS300 software built into a Carl Zeiss image analyzer, the areas were measured and expressed in square micrometers (*μ*m^2^).

### 2.10. Statistical Analysis

Statistical analysis was performed with Student's *t*-test using the software package GraphPad Prism (La Jolla, Calif, USA).

## 3. Results

### 3.1. Purification of GPI-Anchored Proteins

**Figure 1:**
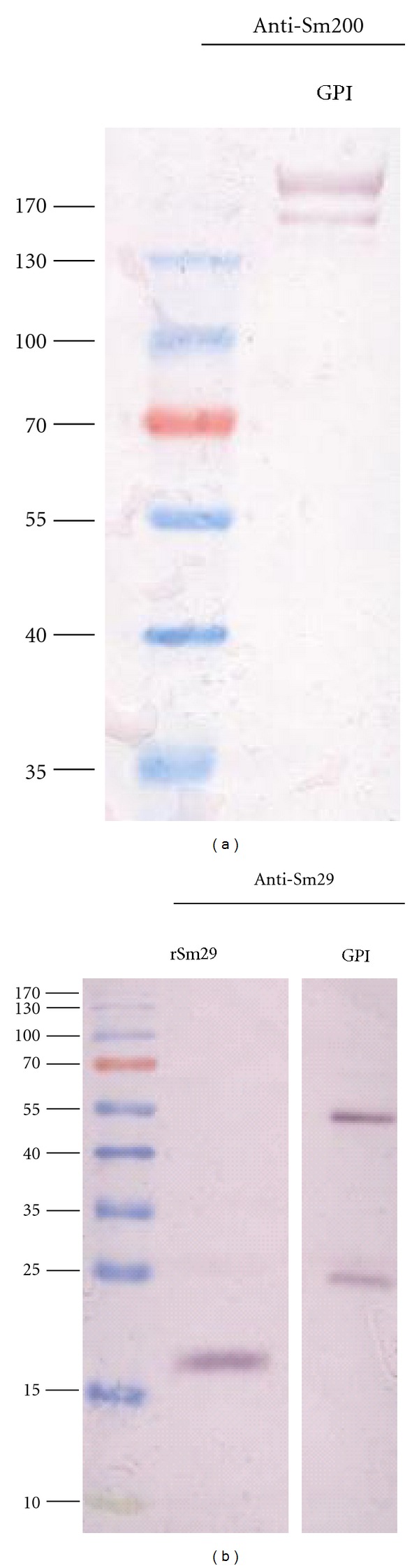
Analysis of GPI-anchored proteins purified from the outer surface of adult *S. mansoni* worms. (a) Western blot analysis of GPI-anchored protein preparation (GPI lane) probed with polyclonal mouse anti-Sm200 antibodies. (b) Western blot analysis of GPI-anchored proteins preparation (GPI lane) and recombinant Sm29 (rSm29 lane) probed with polyclonal mouse anti-Sm29 antibodies. The broad range pre-stained ladder from BioRad was used as molecular weight protein standard.

The enrichment for GPI-anchored proteins from the outer surface of live adult worms was achieved through an enzymatic shaving procedure using the Pi-PLC enzyme. The efficiency of this enzymatic treatment was confirmed by checking the presence of two major GPI-anchored proteins known to be present on the outer surface of the tegument, Sm200 and Sm29. By western blot analysis using mouse polyclonal antibodies raised against Sm200 and Sm29, we demonstrated the presence of two bands around 200 kDa (Figure 1(a)) which correspond to the profile expected for the protein Sm200 as earlier shown [12]. The presence of Sm29 in the GPI-anchored protein samples was indicated by two bands, one with 26 kDa and another with 55 kDa, the size expected for the native Sm29 in SDS-PAGE and its dimerized form, respectively (Figure 1(b)). One band of around 18 kDa confirms the size of a recombinant rSm29 used here as a control (Figure 1(b)).

### 3.2. Antibody Profile Following Mouse Immunization

**Figure 2:**
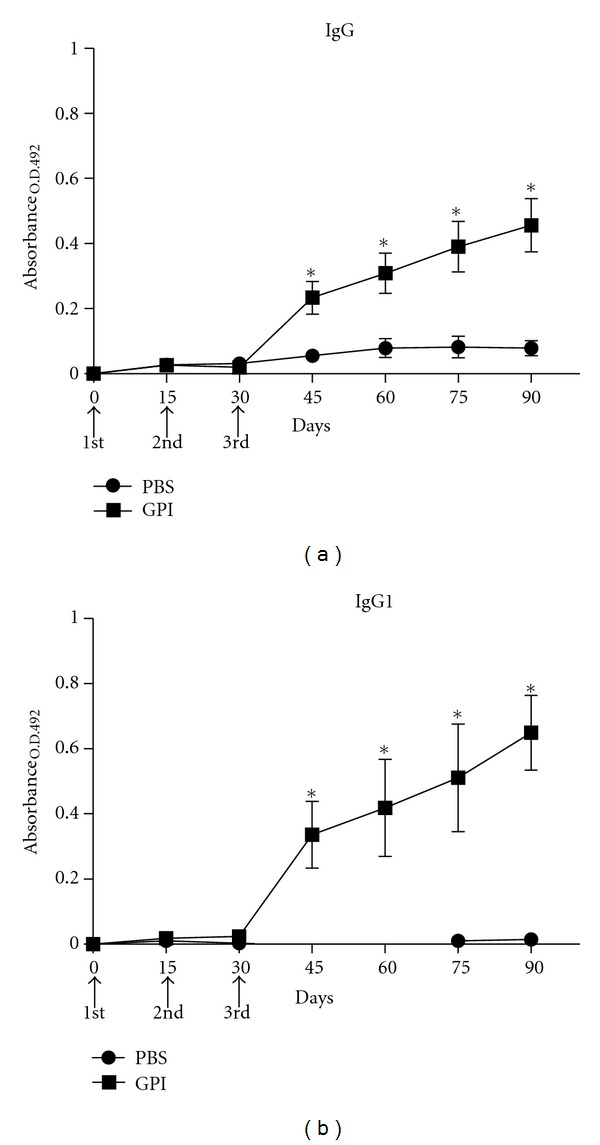
Kinetics of specific IgG and IgG1 anti-GPI-Proteins in sera of mice vaccinated with GPI-Proteins preparations. Sera of 10 immunized mice per group were collected prior first immunization and at days 15, 30, 45, 60, 75, and 90 after first immunization and assayed by ELISA for IgG (a) and IgG1 (b) antibodies. Control group was injected with PBS plus Freund adjuvant. Arrows indicate times of three immunizations. Results are presented as the mean absorbance measured at 492 nm for each group per day of sera collection. Asterisks indicate statistically significant differences of vaccinated groups compared to control group (*P* < 0.05). Results are representative of two independent biological experiments.

The levels of specific antibodies generated against the purified GPI-anchored proteins were determined by ELISA in the sera from ten vaccinated mice of each experimental group. After the third immunization, the titer of specific anti-GPI-anchored proteins IgG antibodies started to increase and was sustained up to day 90 (Figure 2(a)). A similar profile was observed for the levels of the IgG1 subclass, which showed a noticeable increase after the third immunization. The increase of IgG1 titer in the sera of immunized mice was evident up to day 90 (Figure 2(b)). Moreover, there was no increase in the levels of the IgG2a subclass compared to the unvaccinated group throughout the period evaluated in the experiments (data not shown).

**Figure 3:**
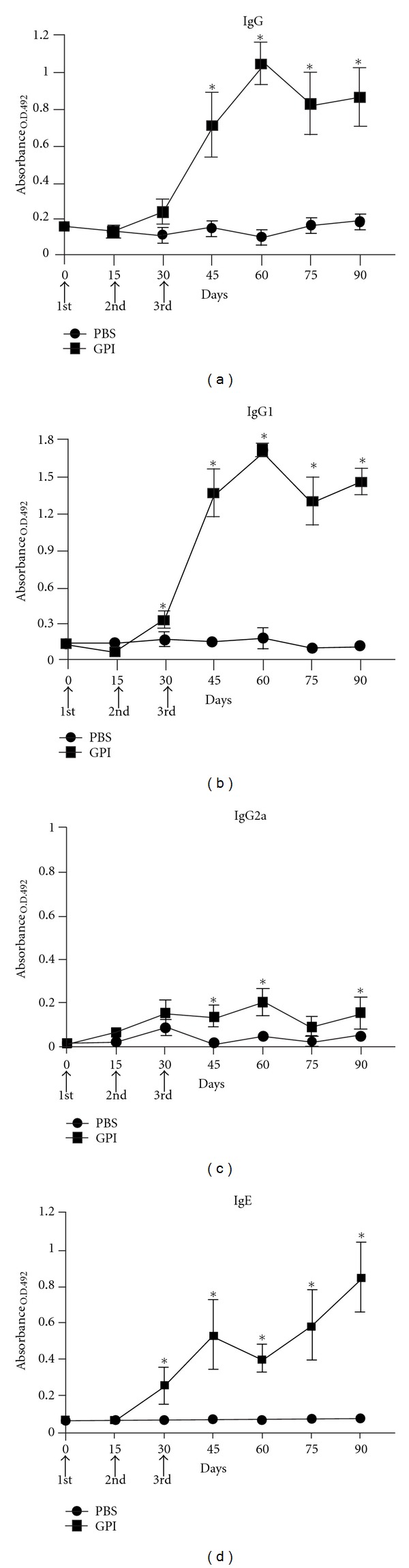
Kinetics of specific antibodies anti-Sm200/Sm29 in sera of mice vaccinated with GPI-Proteins preparations. Sera of 10 immunized mice per group were collected prior first immunization and at days 15, 30, 45, 60, 75 and 90 after first immunization and assayed by ELISA for IgG (a), IgG1 (b), IgG2a (c), and IgE antibodies (d). Control group was injected with PBS plus Freund adjuvant. Arrows indicate times of three immunizations. Results are presented as the mean absorbance measured for each group per day of sera collection. Asterisks indicate statistically significant differences of vaccinated groups compared to control group (*P* < 0.05). Results are representative of two independent biological experiments.

The levels of specific IgG, IgG1, IgG2a, and IgE antibodies against the proteins Sm200 and Sm29 were evaluated in the sera of mice vaccinated with GPI-anchored proteins compared to the control group. Specific anti-Sm200/Sm29 IgG, IgG1, and IgE antibodies were detected after the third immunization and significant increase was observed after 45 days postimmunization (Figure 3). The levels of specific IgG2a in the vaccinated group were significantly increased at 45, 60, and 90 days compared to control group. However, the levels of anti-Sm200/Sm29 IgG2a were not that high compared to nonvaccinated animals.

We also performed the measured of specific antibodies for each of this proteins individually (data not shown). We concluded that the elevated levels of IgG1 and IgE observed in Figure 3 were mostly due to Sm200 and not Sm29.

### 3.3. Cytokine Profile

**Figure 4:**
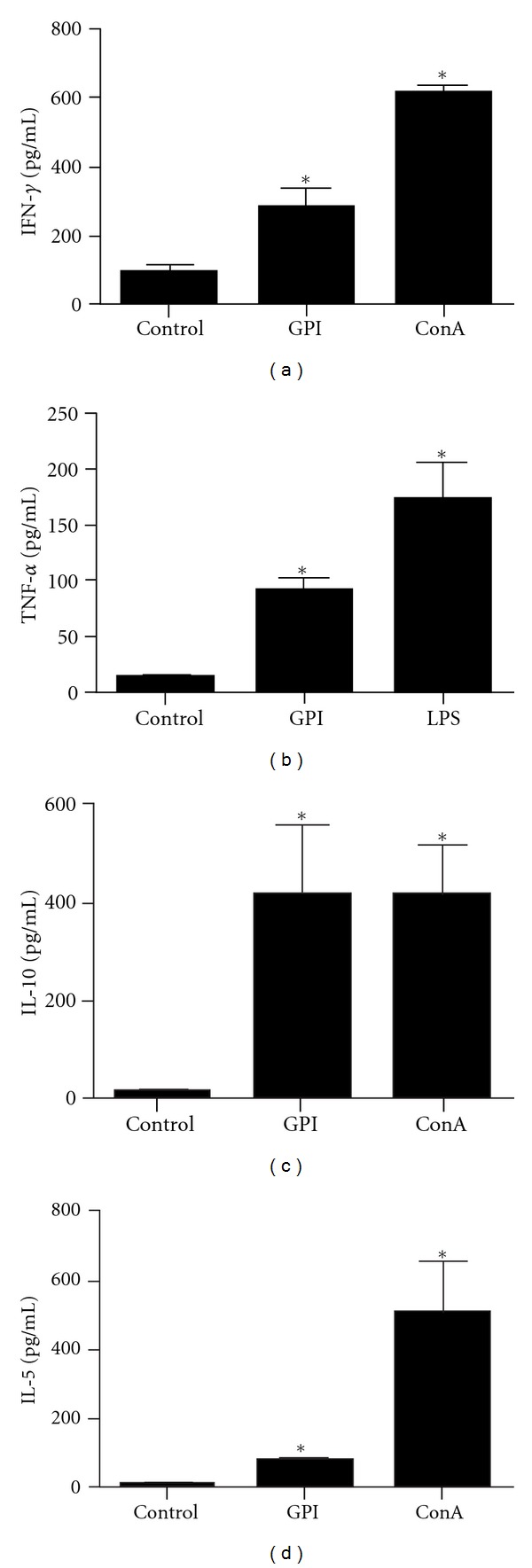
Cytokine profile of mice immunized with GPI-Proteins. Control group was immunized with PBS plus Freund adjuvant (CFA/IFA). One week after the last immunization, splenocytes from five mice were isolated and assayed for, IFN-*γ*, TNF-*α*, IL10 and IL5 production in response to GPI proteins (12.5 *μ*g/mL + CFA/IFA), or PBS + CFA/IFA as control. The results are presented as mean ± S.D. for each group. A statistically significant difference of GPI vaccinated mice compared to control group is denoted by an asterisk for *P* < 0.05. Concanavalin A (ConA) and LPS were used as positive controls. Results are representative of two independent biological experiments.

To evaluate the cytokine profile generated by the immunization of mice with GPI-anchored proteins, splenocytes were isolated from spleens of vaccinated and control animals after the third immunization. Statistically significant levels of IFN-*γ* (320 pg/mL) and TNF-*α* (95 pg/mL), proinflammatory cytokines and characteristic of Th1-type of immune response, were produced by splenocytes of immunized group compared to the control group (Figures 4(a) and 4(b)). Additionally, significant amounts of IL-5 (78 pg/mL), a signature of Th2-immune response, and IL-10 (425 pg/mL), a regulatory cytokine, were detected in samples of vaccinated mice compared to control group (Figures 4(c) and 4(d)). Concanavalin A (625 pg/mL-IFN-*γ*, 408 pg/mL-IL-10, 515 pg/mL-IL-5) and LPS (172 pg/mL-TNF-*α*) were used as positive controls.

### 3.4. Worm Burden Recovery

The protective immunity induced by GPI-anchored protein vaccination was analyzed 45 days after challenge infection with 100 cercariae per mouse. Immunized mice showed significant reduction on adult worms burden recovered from the mesenteric veins when compared to the control group. Similar results were observed in two independent experiments. Vaccination with GPI-anchored proteins engendered 42% of reduction in adult worm burden (Table 1).

### 3.5. Reduction in Liver Pathology

**Figure 5:**
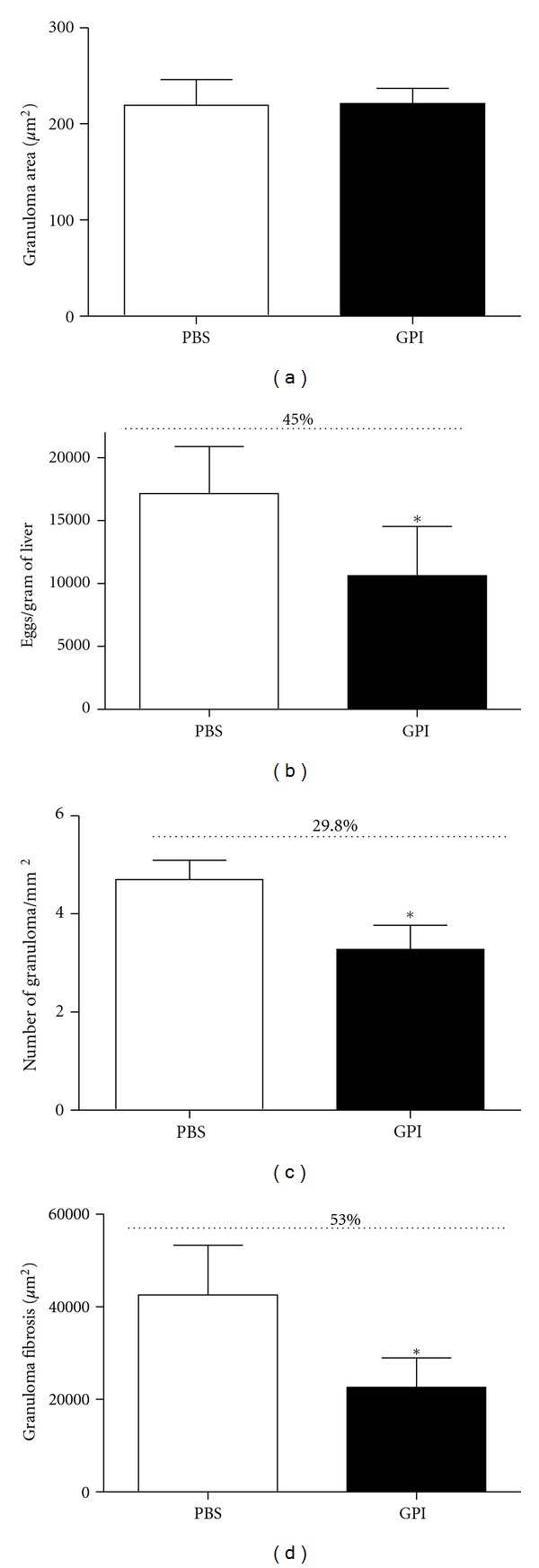
Liver pathology measured in mice vaccinated with GPI-anchored proteins. (a) Hepatic granuloma area, (b) Number of eggs per gram of hepatic tissue, (c) hepatic granuloma numbers, and (d) fibrosis area. The quantification of eggs and the number of granulomas in histological sections were performed using a microscope with 10x objective lens. The results are expressed in square micrometer for granuloma area, in eggs per gram of tissue for number of eggs, in granuloma per square milimeter for number of granuloma and fibrosis per square micrometer for granuloma fibrosis. A statistically significant difference of GPI vaccinated mice compared to control group is denoted by an asterisk for *P* < 0.05. Results are representative of two independent biological experiments.

**Figure 6:**
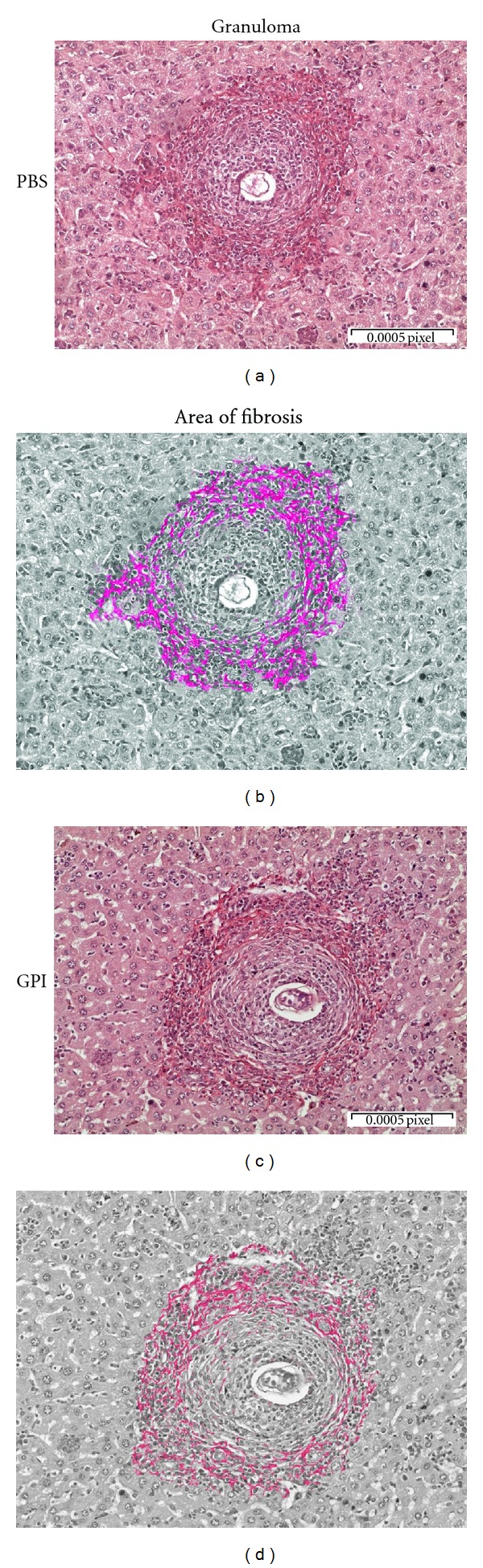
Histological analysis of hepatic tissue from mice vaccinated with GPI-anchored proteins. Animals were sacrificed and their livers were washed in PBS and stored in formaldehyde until sectioning and staining with Picrosirius. (a) Representative sample from PBS control group. (c) Representative sample from mice vaccinated with GPI-anchored proteins. ((b) and (d)) Images were edited using image software to highlight in red the area of fibrosis. Images were capture in 40x objective lens and they are representative of two independent biological experiments.

The consequence of GPI-anchored protein vaccination for liver pathology was evaluated by histological analysis of fixed and stained sections of hepatic tissue, as well as by quantification of eggs per gram of hepatic tissue. Immunized mice had 45% reduction in eggs per gram of tissue (Figure 5(b)), 29% reduction in the number of granulomas per area (Figure 5(c)), and 53% reduction in the granuloma fibrosis (Figure 5(d)). However, no difference was observed regarding the area of the granulomas between vaccinated and control groups (Figure 5(a)). The granuloma and area of fibrosis can be visualized on Figure 6.

## 4. Discussion

Schistosomiasis is one of the most important neglected tropical diseases for which an effective control is unlikely in the absence of improved sanitation and a vaccine. As mentioned earlier, the tegument is a promising source of potential targets for the development of an antischistosomiasis vaccine. In a recent publication, it was reported that a preparation containing tegument antigens extracted from *S. mansoni* schistosomula when used as vaccine in mice elicited reduction in the number of eggs trapped in the liver, the number of eggs eliminated in the faeces, the number of hepatic granulomas and parasite burden [19]. However, the work by Teixeira de Melo et al. [19] is different from our study since we are using here only GPI-anchored proteins and not the entire tegument. Several tegument and tegument-associated proteins have been evaluated as vaccine candidates in the last years, such as Sm29 [9], TSP2 [8], Sm22.6 [23], a Stomatin-Like Protein-2 [24], Sm-p80 (a subunit of calpain) [25], ECL (Sm200) [26], Sm25 [27, 28] among many others (for a better review see Pinheiro et al. 2011 [29]). Even with many attempts using tegument proteins as subunit vaccines in the form of recombinant proteins or as DNA vaccines, up to now, no single vaccine candidate achieved the protection levels obtained by radiation attenuated cercariae [30]. Regarding protein localization among all tegument proteins, there are some not surface exposed and others that are tegument membrane bound such as Sm29, Sm200 and TSP2. This last protein is bound to the membrane by transmembrane domains, while Sm29 and Sm200 are GPI-anchored to the outer surface of the membrane as previously demonstrated [12, 13]. The characteristic of a protein be surface exposed increases the probabilities of interactions with the mammalian host as well as the accessibility to antibodies and cells of the immune system. In this study, we have used an enzymatic approach to enrich for GPI-anchored proteins from the outer surface of live adult *S. mansoni *worms (males and females) and confirmed the presence of the tegument proteins Sm29 and Sm200 (Figure 1). Recently, it was demonstrated that this protocol produces a mixture of proteins in which Sm29 and Sm200 are among GPI-anchored molecules on the outer surface of *S. mansoni* adult worms [13]. The vaccination of mice with this GPI-anchored protein preparation-induced protection in vaccinated mice with 42% of worm burden reduction (Table 1). It is known that immunization with recombinant Sm29 is able to induce a polarized Th1 protective response in mice, with increased levels of IgG, IgG1, and IgG2a anti-Sm29 [9]. Additionally, the immunization of mice with DNA vaccine encoding the gene of Sm200 (ECL) also elicited high levels of IgG, IgG1 and low levels of IgG2a and conferred 33–44% of protection [31]. Moreover, the stimulation of splenocytes with GPI-anchored proteins resulted in increased levels of IFN-*γ*, TNF-*α*, and IL-5. Thus, the cytokine profile elicited by GPI-anchored proteins suggests a mixed Th1/Th2 type of immune response characterized by elevated levels of IFN-*γ* and TNF-*α* (Th1) and significant amounts of IL-5 (Th2). Despite the mixed type of immune response elicited by GPI-anchored protein vaccination, we observed protection in vaccinated mice with 42% of worm burden reduction. The involvement of IFN-*γ* in the protective response maybe related to the capacity of this molecule to elicit an efficient response in the pulmonary region against the migrating parasite [32]. Regarding the role of TNF-*α* in protective responses to *S. mansoni,* it is probably due to the ability of this cytokine in inducing the expression of NO (nitric oxide). The absence of the molecule TNFRI (tumour-necrosis factor receptor-1) in mice immunized with irradiated cercariae is responsible for reducing the level of protection achieved [33, 34].

Regarding liver pathology, GPI-anchored protein vaccination engendered 45% reduction in eggs per gram of tissue, 29% reduction in the number of granulomas per area, and 53% reduction in the granuloma fibrosis. In previous studies performed by our group the majority of *S. mansoni* antigens tested as recombinant protein vaccines that conferred partial protection had induced a Th1 type of immune response [9, 22, 35]. Nevertheless, the recombinant protein rSm22.6-induced partial protection against challenge infection (34%) regardless the mixed type of immune response (Th1/Th2) engendered by this candidate vaccine [23]. Filamin is another protein that has the ability to elicit a mixed type of immune response in mice, which in combination with high titers of IgG, IgG1, IgG2a, and IgG2b is responsible for 44–57% of worms reduction [36]. In another study, protection induced by vaccination with Sb14-3-3zeta antigen was achieved independently of a Th1 type of immune response [37]. The reduction in adult worm burden observed in GPI-anchored vaccinated animals can be directly associated to the reduction in the number of eggs trapped in the liver. Regarding the liver pathology, elevated levels of IL-10 observed in immunized mice might be regulating Th2 responses and/or preventing the development of polarized Th1 responses and consequently, reducing inflammation and liver injury [38, 39].

 In conclusion, our study demonstrated that a preparation containing GPI-anchored proteins that are exposed on the outer surface of adult *S. mansoni* worms engendered a mixed Th1/Th2 type of immune response in mice, which conferred protection against cercarial challenge, and reduced liver pathology. Although Sm29 and Sm200 are among the GPI-anchored proteins present in this preparation, we cannot rule out the possibility that other molecules might be acting synergistically to generate protection against challenge infection. In fact, the proteomic characterization of this GPI-anchored fraction revealed the presence of additional 5 GPI-anchored molecules of parasite origin, among them alkaline phosphatase, CD59 orthologues, and carbonic anhydrase plus a number of other soluble constituents derived from the underlying syncytium and parasite gut [12]. Whether these other components contributed to produce the mixed-type immune response observed remains to be determined. Future protocols designed to obtain highly purified GPI-anchored proteins from the parasite surface will allow full exploration of the potential of the GPI-fraction as a vaccine preparation.

 Finally, even though developing a vaccine against schistosomiasis is a challenging endeavor, the evidence that humans acquire immunity to schistosomes together with new information obtained from genomics and proteomics approaches open an avenue of opportunity to be explored by scientists of developing countries.

## Figures and Tables

**Table 1:** Worm burden reduction and protection level induced in mice vaccinated with GPI-anchored proteins.

Groups	Worm burden (Mean ± S.D.)	Protection
PBS + CFA/IFA	46.08 ± 4.3	
GPI + CFA/IFA	26.58 ± 4.2	42.31%*

CFA: complete Freund's adjuvant; IFA: incomplete Freund's adjuvant. Statistical analyses were performed with Student's *t*-test, *statistically significant (*P*< 0.05) compared to control group. Data are representative of two independent biological assays.
